# Effects of 3d transition metal impurities and vacancy defects on electronic and magnetic properties of pentagonal Pd_2_S_4_: competition between exchange splitting and crystal fields

**DOI:** 10.1038/s41598-022-14780-z

**Published:** 2022-06-27

**Authors:** Mojtaba Gholami, Zahra Golsanamlou, H. Rahimpour Soleimani

**Affiliations:** 1 Department of Physics, University Campus2, University of Guilan, Rasht, Iran; 2 Computational Nanophysics Laboratory (CNL), Department of Physics, University of Guilan, P. O. Box 41335‑1914, Rasht, Iran

**Keywords:** Nanoscience and technology, Physics

## Abstract

In this paper, we first investigate the electronic properties of the two-dimensional structure of dichalcogenide Pd_2_S_4_. These properties strongly depend on the crystal field splitting which can change by atomic vacancies (S and Pd vacancies). The main purpose of the present paper is to create remarkable magnetic properties in the system by adding 3d transition metal atoms where the presence of Mn, Cr, and Fe creates the exchange interaction in the system as well as change in the crystal field. The created magnetic properties strongly depend on the competition between exchange interaction and crystal field to separate the levels of d orbitals. In addition, the presence of the transition metals in the structures with S and Pd vacancy has been investigated carefully. The calculations demonstrate that we can achieve an extensive range of magnetic moment up to 3.131 ${\mu }_{B}$. The maximum one is obtained in the presence of Mn and absence of sulfur while some of the doped structures does not have magnetic moment. Our results show that Pd vacancy in the presence of Cr, Mn and Fe metals increases the magnetic property of the Pd_2_S_4_ structure. The extensiveness and variety of the obtained properties can be used for different magnetic and non-magnetic applications.

## Introduction

The two-dimensional (2D) materials has received great interest for their splendid physical properties such as charge mobility, mechanical, electrical and transport properties^1–5^.

In addition to the intrinsic properties of 2D material, the changing and improving the electrical and magnetic properties of them using molecular, atomic and linear defects are mostly investigated^6–9^.

Among 2D materials, transition-metal dichalcogenides (TMDs) are vastly studied and analyzed recently. Most of these materials have unique 2D characteristics that have never been observed in three-dimensional structures^10–13^. TMDs mostly form an essential group of multi-layer materials with a general formula of MX_2_. In this formula, M and X represent a transition material and a chalcogenide atom, respectively^14^. These materials are comprised of 2D layers where each layer includes three metal atoms and one chalcogen atom located between the metal atom. The atoms are connected with weak covalent bonds. TMD structures have unique chemical and impressive physical characteristics due to their chemical bond conditions^15^.

One of the interesting features to investigate is the magnetic properties of TMDs. Most of these materials do not hold magnetic features in their initial synthesized form, despite their remarkable electronic and mechanical properties. Since many novel applications can be achieved by adding magnetic characteristics to these 2D structures, extensive studies and research are performed in this field^16–27^.

The regular methods to study the magnetic properties are based on the topology method, applying strain forces, vacuuming, and atomic vacancy defect generation. In addition, one of the most significant magnetization methods is doping the structure with transition elements of different periods of the periodic table^28–38^. Ataca et al.^28^ created the T and H-phase conditions on an extensive range of TMDs and investigated the magnetization conditions on the structures. They revealed that VX_2_ (where X could be O, S, Se, or Te) is magnetized at each of the two phases, while NiX_2_ is not. They also demonstrated that the magnetization of ScX_2_ depends on the phase type. The magnetic characteristics of MoS_2_ are studied by creating defects and vacuuming by Wang et al.^29^ They observed that magnetizing of the structure could not be achieved only by vacuuming. Therefore, they combined the vacuuming method by doping with transition metals such as Mn, Fe, and Co and clearly, observed the magnetic properties of the system.

Applying strain is one of the other magnetization methods. The work was performed by Lv et al. can be noted in this regard^30^. They revealed that a gradual increase of strain magnetizes 2D CrSe_2_ and CsTe_2_ and generates ferromagnetic and anti-ferromagnetic properties in the structures.

As mentioned earlier, creating an atomic vacancy defect is another technique for TMD magnetization which can be obtained by eliminating of one or some of the atoms in the structure. Avsar et al.^31^ studied the magnetization of a PtSe_2_ structure by removing Se and Pt atoms. Their results revealed that a Pt vacancy increases the magnetization of the system by 1.2 ${\upmu }_{\mathrm{B}}$. In contrast, a Se vacancy does not produce any magnetization effects. Ma et al. investigated the MoTe_2_ and MoSe_2_ structures by applying defects and removing Mo atoms^32^. Their study demonstrated that only MoSe_2_ structures have spin polarization and magnetic properties. In another work, Gao et al. observed that MoSe_2_ structures do not acquire magnetic characteristics by applying S vacancy defects^33^.

One of the common and accessible approaches to magnetizing the structures is the doping of TMDs. There have been numerous studies in this field. It has to be mentioned that in these works the majority of dopants are transition metals. Zhang et al. doped the 2D metal (CrS_2_) with transition metals and some alkaline earth elements like Ca and demonstrated that the dopants except vanadium can magnetize the structure^34^. In another work, Yue et al. found that the acquired magnetization of MoS_2_, when doped by F, N, B, and H elements, is similar and has a value of 1.0 ${\upmu }_{\mathrm{B}}$, while the magnetic influence of Co, Fe, Mn, Cr, and V elements on MoS_2_ are different^35^. In similar research, Tedstone et al. studied the effects of MoS_2_ doping with 3d transition metals and resulted that Fe, Mn, and Co elements provide the maximum magnetic intensities in their doped structures^36^. ZrS_2_ was another TMD which doped by transition elements and alkaline metals^37^. The results demonstrated that Mn and Cr elements have the greatest magnetic influence, while Ni and Ti have no role in the system magnetization. Apart 3d metal dopants, the role of 4d transition metals for magnetization of TMDs like WSe_2_ were investigated by Hashemi et al. This new examination demonstrated the magnetization of the structure using Zr, Nb, Mo, and Tc dopants. However, any magnetic effect was not obtained in the structures doped by Y, Ru, Rh, and Pd elements^38^.

Among TMDs, Pd_2_S_4_ is one of the fascinating 2D structures. It is composed of two palladium atoms and four sulfur atoms and can be viewed as an intrinsic semiconductor^39^. This structure is a member of 2D pentagonal TM_2_X_4_ materials (where TM is the transition metal and X can be S, Se, or Te) despite the other TMDs that are usually quadrangular or hexagonal. It transforms easily from a semiconductor to a conductor under a particular metamorphosis (e.g., stress) and turns into sheet form from a bulk state^40^. This transformation capability can be the origin of some exciting electronic and photonic characteristics. In fact, it has unique anisotropic features with band gap of almost 1.2 eV which is indirect gap. However, it has a gap in 1 T-phase and a zero-gap in bulk phase. The size of gap changes in multilayer structure where the two-layer structure of Pd_2_S_4_ has a gap of 0.952 eV^41–44^. There has also been done some research on the edges of the Pd_2_S_4_. This structure has different electric properties in nanoribbon mode compared with the bulk mode, so that exhibits a metal-like behavior in zigzag edge shape and semiconductor behavior in armchair one. Although there are extensive research articles on Pd_2_S_4_ in the fields of electronics and magnetism, they have not considered properly the doping of Pd_2_S_4_^45–48^.

In this paper, the magnetic properties of the Pd_2_S_4_ monolayer^49^ are studied through doping with 3d transition metals (Sc, Ti, V, Cr, Mn, Fe, Co, Ni, Cu, and Zn) and creating a vacancy defect by removing a Pd atom and S atom. To the best of our knowledge, there is no published research article on doped Pd_2_S_4_ structure by 3d transition metal in the presence of Pd and S vacancies. Firstly, the spin behaviors of the orbitals of the valence bands of S and Pd atoms are analyzed. Furthermore, their behaviors at the valence and conduction bands boundary are studied without considering the doping and vacancy effects on the structure. Then, the impact of Pd and S vacancies are examined, and the achieved results are analyzed. Finally, to conduct a more comprehensive study, situations with and without atomic vacancy are considered in the presence of transition metal dopants. In addition, the effects of 3d metal doping and atomic vacancy on the band gap are investigated. The simultaneous evaluation of the effects of doping and defect on the material is one of the considerable aspects of this work. It is demonstrated that doping has more influence on magnetization than atomic vacancies. In other words, doping with some elements leads to magnetization, while doping with some other elements causes no magnetic effects. Moreover, both doping and vacancies have significant roles in reducing the band gap and changing its type.

## Methods

**Figure 1:**
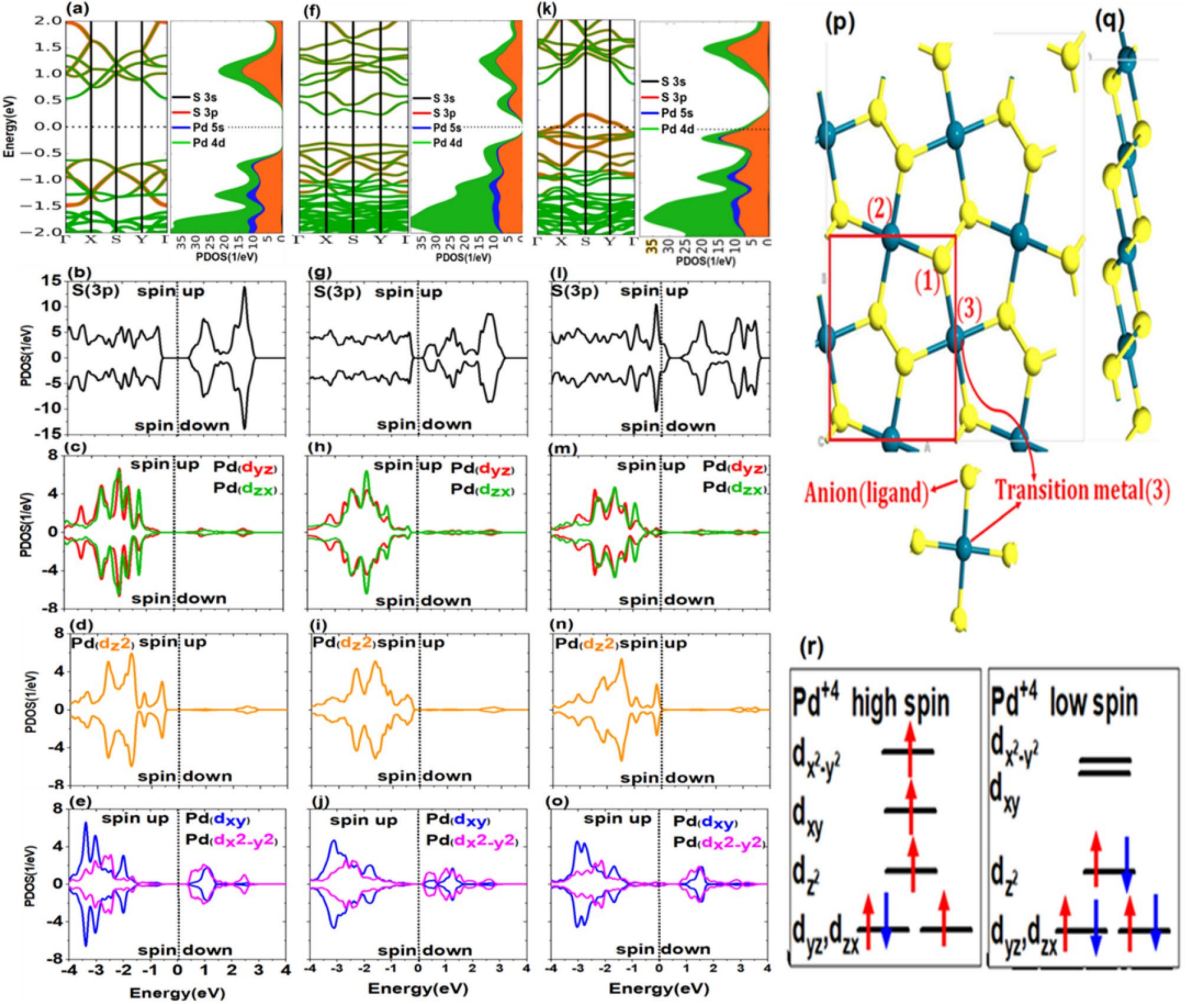
Band structure and density of states for 3p orbitals of S atom and 4d orbitals of Pd atom in Pd_2_S_4_ structure: (**a**–**e**) without vacancy defect, (**f**–**j**) arising from defects in S atoms, (**k**–**o**) arising from defects in Pd atoms, (**p**) top view of Pd_2_S_4_ structure with the position of vacancy defect for S (No. 1) and Pd (No. 2) atoms, (**q**) side view of Pd_2_S_4_ structure, and (**r**) electrons located in 4d orbitals of Pd^+4 ^ cation in strong and weak crystal fields.

Our Density functional theory (DFT) calculations have been employed based on the Perdew, Burke and Ernzerhof (PBE)^50^ variant of the exchange–correlation functional and the projector augmented wave (PAW) pseudopotentials^51^, as implemented in Siesta^52^. The spin dependent non-relativistic version of SGGA is used for correlation-equilibrium potential and GGA is used for non-polarization mode. The set of basic states used for the valences of TM-3d, S and Pd atoms are (3s 3p 4s 3d), (3s 3p), and (4s 3d 4p 4d), respectively. To find the equilibrium point in the calculations and the accuracy in calculating the atomic force, the relaxation value reached less than 0.05 eV/Å and also the accuracy of the calculations has been considered as 10^–5^ eV. In evaluation related to magnetic state and the relaxation, the Brillouin zone was sampled with a 40 × 40 × 1 and 14 × 14 × 1, respectively. These compounds are composed of $\mathrm{S}-\mathrm{Pd}-\mathrm{S}-\mathrm{S}-\mathrm{Pd}-\mathrm{S}$ planes where $\mathrm{Pd}-\mathrm{S}$ and $\mathrm{S}-\mathrm{S}$ are ionic and covalent bonds, respectively. Figure 1 illustrates the top view (p) and side view (q) of a $2\times 2$ supercell of ${\mathrm{Pd}}_{2}{\mathrm{S}}_{4}$ monolayer. As it can be seen, the positioning of Pd atoms in this structure is such that every Pd atom is in contact with four S atoms. Each pentagonal ring of ${\mathrm{Pd}}_{2}{\mathrm{S}}_{4}$ includes four $\mathrm{Pd}-\mathrm{S}$ bonds and an $\mathrm{S}-\mathrm{S}$ bond. We construct a 2 × 2 × 1 supercell based on the primitive cell, as shown in Fig. 1p,q. A vacuum region about 12 A is used to for decoupling between neighboring layers. This supercell contains 8 Pd and 16 S atoms respectively. The calculated bond lengths for Pd–S and TM-S atoms are 2.358 Å and 2.332 Å, respectively. To study the electric and magnetic characteristics of doped TMD, the LBFGS-optimized structures were used. Besides tolerable force and pressure are assumed to be 0.05 eV/Å and 0.2GPa, respectively.

## Results

### Electronic properties of monolayer $\mathbf{P}{\mathbf{d}}_{2}{\mathbf{S}}_{4}$

The ${\mathrm{Pd}}_{2}{\mathrm{S}}_{4}$ monolayer belongs to a simple orthorhombic crystal lattice structure with space group *Pbca* (P2_1/c) and point group (2/m)^49^. According to the results of the phonon dispersion of the ${\mathrm{Pd}}_{2}{\mathrm{S}}_{4}$ monolayer, presented by M. N. Gjerding et al., this structure is stable^49^. Absence of negative vibrational modes to be the main parameters determining the stability of the ${\mathrm{Pd}}_{2}{\mathrm{S}}_{4}$ monolayer. We expect that the replacement of only one of Pd atoms in the large supercell cell, which is considered in the calculations, with another transition metals atom will not have significant changes in system stability.

After optimizing the system, computations indicate that the lattice constants and bond length between a Pd atom and nearest S atoms are $a=5.4833$, $b=5.4833$, $c=16.3359$, and ${d}_{Pd-s}=3.433$ Å. Also, the covalent bond $\mathrm{S}-\mathrm{S}$ has a length of ${d}_{s-s}=2.12$ Å. Concerning the crucial importance of valence electrons in establishing the electronic and magnetic properties, valence orbitals, $\mathrm{Pd}(5{\mathrm{s}}^{0 },4{\mathrm{d}}^{10})$ and $\mathrm{S}(3{\mathrm{s}}^{2},3{\mathrm{p}}^{4})$, play an essential role in structural hybridization.

Figure 1a shows the electronic structure and the contribution of the valence orbitals of S and Pd atoms to the band structure and projected density of states (PDOS). As shown in Fig. 1a, the d orbitals of the Pd and p orbitals of the S have significant contributions in structure formation. These valence and conduction bands orbitals exhibit a complete overlap and proper hybridization. The two 3p and 4d orbitals in S and Pd atoms, respectively, have more contribution in the valence band maximum (VBM) and conduction band minimum (CBM) of the structure. These orbitals are the linking bridges between ligands and metals in ${\mathrm{Pd}}_{2}{\mathrm{S}}_{4}$.

Figure 1a also reveals that 3s orbitals in S atoms and 5s orbitals in Pd atoms have the lowest PDOS contributions to the valence and conduction bands and cannot play an essential role in the electronic structure. In the following, we generally concentrate our investigation on 3p orbitals in S atoms and 4d orbitals in Pd atoms.

In a straightforward and approximate embodiment, it can be stated that when the S atom is present in the monolayer structure, it obtains two electrons from the adjacent Pd atoms due to the higher electronegativity than Pd. Because of being present next to four S atoms, the Pd atom is converted to Pd^4+^ after hybridization. The five orbitals ${\mathrm{d}}_{\mathrm{xy}}$, ${\mathrm{d}}_{\mathrm{yz}}$, ${\mathrm{d}}_{\mathrm{zx}}$, ${\mathrm{d}}_{{\mathrm{z}}^{2}}$, ${\mathrm{d}}_{{\mathrm{x}}^{2}-{\mathrm{y}}^{2}}$ of the Pd atom are split under the effect of the crystal field. The 4d orbitals are greater in extent compared to 3d ones.

In addition, the electronic and magnetic properties of the system are significantly dependent on how the orbitals are filled close to the Fermi level. Considering the evaluations near the Fermi level, the splitting mechanism is demonstrated in Fig. 1a–e regarding the results obtained from the PDOS of 4d orbitals of the Pd atom. Among five orbitals ${\mathrm{d}}_{\mathrm{xy}}$, ${\mathrm{d}}_{\mathrm{yz}}$, ${\mathrm{d}}_{\mathrm{zx}}$, ${\mathrm{d}}_{{\mathrm{z}}^{2}}$, ${\mathrm{d}}_{{\mathrm{x}}^{2}-{\mathrm{y}}^{2}}$ (Fig. 1c–e) of Pd atom, ${\mathrm{d}}_{\mathrm{xy}}$ and ${\mathrm{d}}_{{\mathrm{x}}^{2}-{\mathrm{y}}^{2}}$ (Fig. 1e) have the highest number of vacancy states. Three remaining orbitals have no states at energy levels higher than Fermi energy. Based on the obtained data, also, the crystal field theory, the way that orbitals are positioned and filled can be predicted. Five orbitals of Pd atom located in the range of − 2 eV to + 2 eV. As indicated in Fig. 1r, orbitals can be filled in two ways. In the first state which is high-spin configuration, all spitted d-orbitals almost have electrons. In the second state or low-spin configuration, ${\text{d}}_{{{\text{yz}}}}$ and ${\text{d}}_{{{\text{zx}}}}$ are degenerate orbitals as it can also be found from Fig. 1c. These are the lowest d orbitals in terms of energy level for both high and low spins states and are fully occupied by low spin states. The ${\text{d}}_{{{\text{z}}^{2} }}$ (Fig. 1d) orbital is also full and has an energy level higher than ${\text{d}}_{{{\text{yz}}}}$ and ${\text{d}}_{{{\text{zx}}}}$ (Fig. 1c). However, ${\text{d}}_{{{\text{xy}}}}$ and ${\text{d}}_{{{\text{x}}^{2} - {\text{y}}^{2} }}$ (Fig. 1e) are vacant and have much higher energy levels. Thus being in one of these two states depends on the separation of d-orbital which is affected by the strength of the crystal field. Since the Pd^4+^ structure includes d orbitals, which are higher in energy with respect to s and p orbitals, it overlaps well with p orbitals of adjacent ligand ${\text{S}}^{ - 2}$ in $\left[ {{\text{Pd}}_{2} {\text{S}}_{4} } \right]^{ - 2}$^53^.

As a result, because of pd hybridization between metallic and ligand orbitals, a strong field is created causing d orbitals of Pd to be further split. According to Fig. 1r, if the crystal field is weak, the distance between d-orbital energy levels is less and more unpaired electrons occupy the levels.

Also, if the crystal field is strong, six electrons of Pd^4+^ are located pairwise in ${\mathrm{d}}_{\mathrm{yz}}$, ${\mathrm{d}}_{\mathrm{zx}}$ (Fig. 1c),and ${\mathrm{d}}_{{\mathrm{z}}^{2}}$ (Fig. 1d) orbitals with lower energies and the higher energy orbitals are almost vacant, see Fig. 1e, (low spin) . Therefore, up and down spins of ${\mathrm{d}}_{\mathrm{yz}}$, ${\mathrm{d}}_{\mathrm{zx}}$ (Fig. 1c) and ${\mathrm{d}}_{{\mathrm{z}}^{2}}$ (Fig. 1d) orbitals completely neutralize each other, and the structure will have no magnetic properties. Obtained results show that ${\mathrm{Pd}}_{2}{\mathrm{S}}_{4}$ has no magnetic property, which is consistent with experimental results^42,43^. Thus, in the square planar form, the d orbitals of Pd are split so that four single electrons prefer to be paired and carry the pairing energy than transferring to higher energy orbitals which are ${\mathrm{d}}_{\mathrm{xy}}$ and ${\mathrm{d}}_{{\mathrm{x}}^{2}-{\mathrm{y}}^{2}}$ (Fig. 1e) The ${\mathrm{p}}_{x}$, ${\mathrm{p}}_{\mathrm{y}}$, and ${\mathrm{p}}_{\mathrm{z}}$ (Fig. 1b)orbitals of the S atom experience no significant change in crystal field due to their symmetric geometry and being in the same energy level. The obtained results indicate that ${\mathrm{Pd}}_{2}{\mathrm{S}}_{4}$ monolayer is a nonmagnetic semiconductor with an indirect band gap of 1.2 eV and identical up spin and down spin PDOS (see Fig. 1b–e). In the square planar structure, electrons of Pd^4+^ fill the energy levels from bottom to top. Given that six high-spin and low-spin electrons have filled three first levels, it can be concluded that no spin magnetization occurs in the pure ${\mathrm{Pd}}_{2}{\mathrm{S}}_{4}$ system.

It is clear from the comparison of 4d orbitals of Pd that although ${\mathrm{d}}_{{\mathrm{z}}^{2}}$ (Fig. 1d) has a more considerable contribution than other d orbitals in a specific valence band in the range of − 1.5 eV to − 0.25 eV, it has a minor contribution in the conduction band. The ${\mathrm{d}}_{\mathrm{xy}}$ and ${\mathrm{d}}_{{\mathrm{x}}^{2}-{\mathrm{y}}^{2}}$ (Fig. 1e) orbitals of Pd devotes the highest contribution in the conduction band, but ${\mathrm{d}}_{\mathrm{yz}}$, ${\mathrm{d}}_{\mathrm{zx}}$ (Fig. 1c), and ${\mathrm{d}}_{{\mathrm{z}}^{2}}$ (Fig. 1d) play more critical role in the formation of valence band due to their lower energy level and higher PDOS.

In the following, we investigate the effect of removing S(1) adjacent to the Pd atom, as shown in Fig. 1p. As can be seen, vacancy defect causes the two Pd atoms and one S atom to have a free or suspended band. After removing S, two electrons of Pd which were bonded to the S atoms become free. Therefore, these electrons can create new levels in the band gap region and decrease the gap. Figure 1f–j displays the band structure and PDOS of ${\mathrm{Pd}}_{2}{\mathrm{S}}_{4}$ monolayer with S vacancy, where the band gap of the system decreases from 1.2 to 0.8 eV and gap becomes direct. The VBM does not change its place whereas CBM changes from $\Gamma$ to X. New states, which play the role of electron acceptors and donors and provide more space for the movement of electrons, are formed in the vicinity of the Fermi level. Under the new crystal field effect, splitting d orbitals of Pd and p orbitals of S experience no major difference relative to the pure state. As shown in Fig. 1h, the pair of degenerate orbitals*,* ${\mathrm{d}}_{\mathrm{yz}}$ and ${\mathrm{d}}_{\mathrm{zx}},$ exhibits approximately similar behavior. Also, the ${\mathrm{d}}_{\mathrm{xy}}$ and ${\mathrm{d}}_{{\mathrm{x}}^{2}-{\mathrm{y}}^{2}}$ (Fig. 1j) orbitals have almost similar behavior, while the single orbital ${\mathrm{d}}_{{\mathrm{z}}^{2}}$(Fig. 1i) is different from others. The monolayer ${\mathrm{Pd}}_{2}{\mathrm{S}}_{4}$ maintains its nonmagnetic property because the PDOS of up-spin and down-spin states is symmetrical, as shown in Fig. 1g–j.

A Pd atom is eliminated from the studied structure for a detailed examination of the crystal field. According to Fig. 1p, by removing the Pd atom No. 2, the geometrical symmetry of the system changes and the first four neighboring S atoms will have free bonds in the system. Hence, those four S atoms have a half-filled orbital which their electrons play an important role in the band structure as they do not form any bonds. Then, new states are created within the band gap and near the Fermi level. Also, the new crystal field does not significantly affect the splitting of d orbitals of Pd compared to the pure state. Based on Fig. 1m, the two orbitals ${\mathrm{d}}_{\mathrm{yz}}$ and ${\mathrm{d}}_{\mathrm{zx}}$ show almost the same behavior and are degenerate. Also, ${\mathrm{d}}_{\mathrm{xy}}$ and ${\mathrm{d}}_{{\mathrm{x}}^{2}-{\mathrm{y}}^{2}}$ (Fig. 1o) orbitals have almost consistent diagrams, while the single orbital ${\mathrm{d}}_{{\mathrm{z}}^{2}}$ (Fig. 1n) is different from others. In the valence band, the role of orbitals ${\mathrm{d}}_{{\mathrm{z}}^{2}}$ (Fig. 1n), ${\mathrm{d}}_{\mathrm{yz}}$ and ${\mathrm{d}}_{\mathrm{zx}}$ (Fig. 1m) is more than ${\mathrm{d}}_{\mathrm{xy}}$ and ${\mathrm{d}}_{{\mathrm{x}}^{2}-{\mathrm{y}}^{2}}$ (Fig. 1o), while the inverse situation can be found in the conduction band. According to Fig. 1l–n, removing a Pd atom notably affects the ${\mathrm{p}}_{\mathrm{x}}$, ${\mathrm{p}}_{\mathrm{y}}$, and ${\mathrm{p}}_{\mathrm{z}}$ orbitals of the S atom so that the Fermi level is deeply shifted toward the interior of orbitals and plays a significant role in the conduction band of the structure. In this case, the system retains its nonmagnetic property, because the d orbitals of Pd atom and the p orbitals of S atom have symmetrical PDOS diagrams for the up-spin and the down-spin states.

### Electronic properties of monolayer $\mathbf{P}{\mathbf{d}}_{2}{\mathbf{S}}_{4}$ in the presence of 3d groups

**Figure 2:**
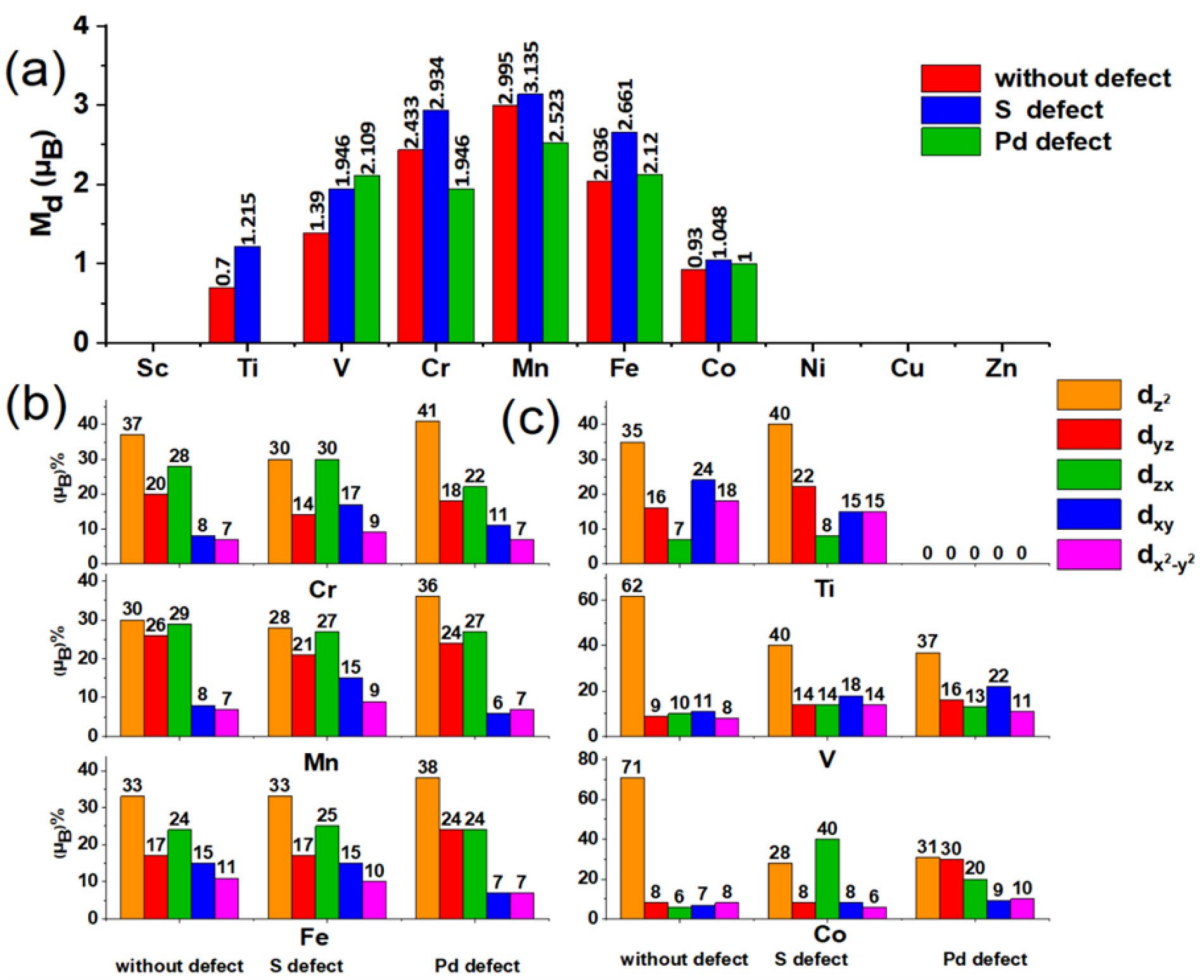
(**a**) Magnetic moment arising from doping of ${\mathrm{Pd}}_{2}{\mathrm{S}}_{4}$ structure with 3d transition metals with and without S and Pd vacancies. (**b**) Percentage of spin separation for five 3d orbitals of the first group (high magnetization) with and without vacancies. (**c**) Percentage of spin separation for five 3d orbitals of the second group (medium magnetization) with and without vacancies.

To create the magnetic property in the structure, as shown in Fig. 1, one of the Pd atoms of ${\mathrm{Pd}}_{2}{\mathrm{S}}_{4}$ are substituted with the metals of 3d group in three different cases. In the first case, the doping occurs in the pure system while in other two cases, the doping is in the presence of Pd or S vacancies. To investigate the doped system, the energy of magnetic (${\mathrm{E}}_{\mathrm{sp}}$) and nonmagnetic states (${\mathrm{E}}_{\mathrm{nsp}}$) due to the doping is calculated and compared among the three cases. The difference between magnetic and nonmagnetic energies of the systems is determined by ${\Delta \mathrm{E}}_{spin}={\mathrm{E}}_{\mathrm{sp}}{-\mathrm{E}}_{\mathrm{nsp}}$. The obtained results for each case are presented in Table S1 of Supplementary Information (SI). The magnetic moment of the doped atom is indicated in Fig. 2. By examining of the obtained results, the effects of 3d transition metals on the magnetization of ${\mathrm{Pd}}_{2}{\mathrm{S}}_{4}$ in considered cases can be divided into three groups: the first includes Mn, Cr, and Fe atoms which has the maximum values of $\Delta {\mathrm{E}}_{\mathrm{spin}}$ and magnetic moment. The second group includes V, Ti, and Co atoms with moderate values of $\Delta {\mathrm{E}}_{\mathrm{spin}}$ and magnetic moment. Finally, the third one involves Sc, Ni, Cu, and Zn atoms where $\Delta {\mathrm{E}}_{\mathrm{spin}}$ is zero. The similar results were obtained for the doping of the 2D materials with transition metals in the previous works^54–57^. To further evaluate, a metal is selected as the representation of each group, and its properties are discussed in more details where, Mn, V, and Ni are examined as the representations of first, second, and third groups, respectively.

Because of the importance of magnetic properties, the first group and Mn atoms are further investigated in this paper. The magnetization (Bohr magneton) of each metal and contributions of first and second neighbors of S and Pd in magnetization are demonstrated in Fig. 2 for three mentioned cases. The outstanding point is that the magnetization for the electrons of d-orbital cannot just be explained based on the splitting of d orbitals according to the square planar model of the pure case (see Fig. 1r). The reason is that the presence of 3d metals results in a strong exchange interaction besides the crystal field. The 3d orbitals of the impurity atoms have a minor spatial extension than 4d orbitals of the Pd atoms and are mainly localized. Therefore, in most cases, there is a competition between these two fields to separate the 3d-orbital energy levels of the impurity, which determines the magnetic properties and moments.

### Electronic and magnetic properties of monolayer $\mathbf{P}{\mathbf{d}}_{2}{\mathbf{S}}_{4}$ in the presence of Mn atoms

The first group and its representation (i.e., Mn) are examined initially. When the Mn with the valence configuration of 3d^5^ 4s^2^ is substituted instead of Pd atom in the structure, it must lose the same electrons as the Pd loses (i.e., four electrons).

**Figure 3:**
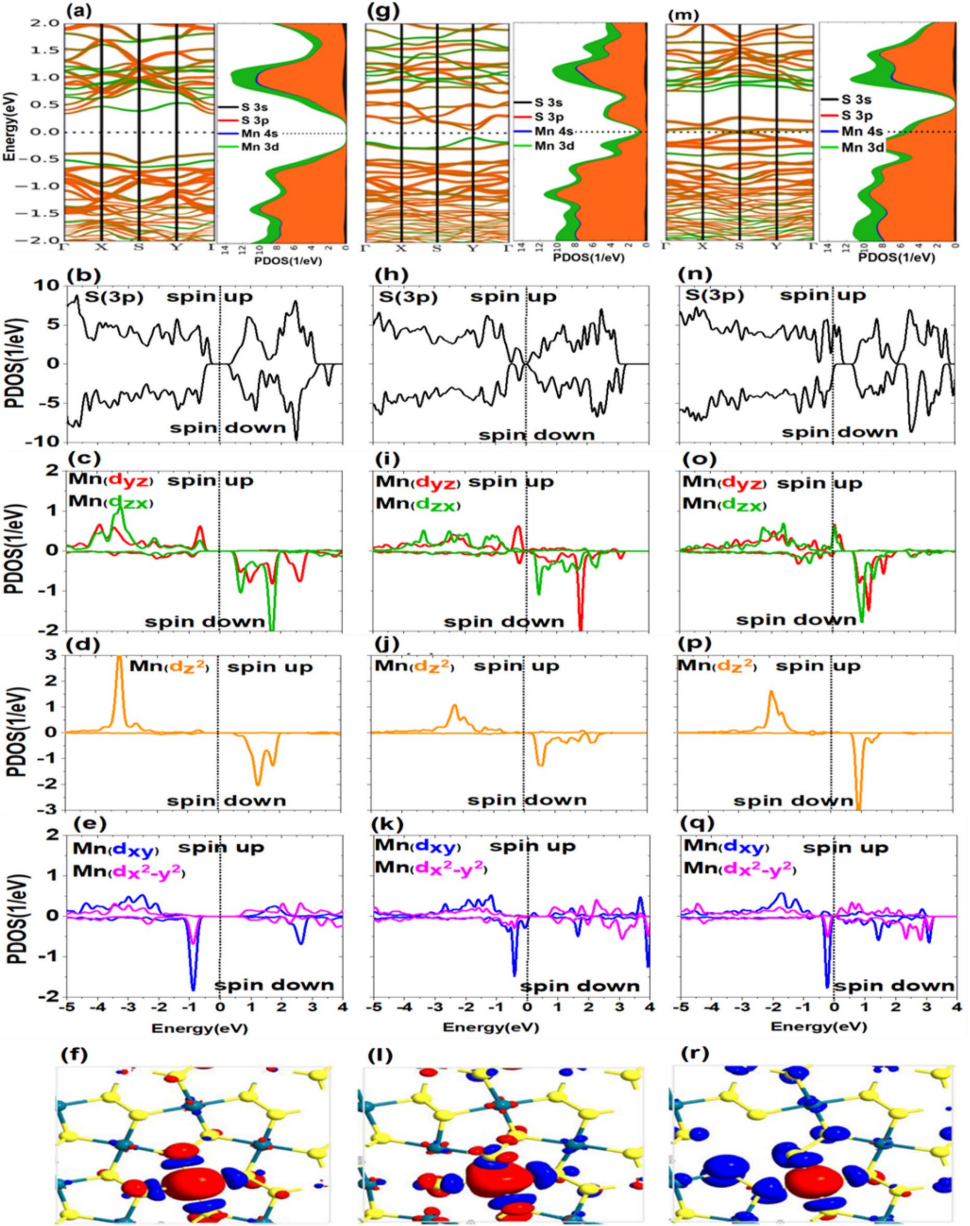
(**a–f**) Band structure and PDOS of p and d orbitals and spin density difference in the presence of transition metal impurity (Mn), (**g–l**) in the presence of the S atom vacancy defect, and (**m–r**) in the presence of the Pd atom vacancy defect.

According to Fig. 2a, the magnetization intensity of Mn is $2.995{\upmu }_{\mathrm{B}}$ in the absence of S and Pd vacancies which is the maximum value among 3d metals impurities. Up-spin and down-spin electrons of 3p orbitals of the S atom are almost symmetrical in this case and play an insignificant role in the magnetic properties of the system. Figure 3a–f shows the PDOS of ${\mathrm{d}}_{\mathrm{yz}}$, ${\mathrm{d}}_{\mathrm{zx}}$, ${\mathrm{d}}_{{\mathrm{z}}^{2}}$, ${\mathrm{d}}_{\mathrm{xy}}$, and ${\mathrm{d}}_{{\mathrm{x}}^{2}-{\mathrm{y}}^{2}}$ orbitals in the presence of Mn without vacancy defect. In this case, the spin separation of the system or exchange interaction is strong. As shown in Fig. 3d, ${\mathrm{d}}_{{\mathrm{z}}^{2}}$ has the maximum localization and minimum bandwidth. The spin separation of ${\mathrm{d}}_{{\mathrm{z}}^{2}}$ is considerable and has profound effects on the magnetic moment of the system. The up-spin and down-spin of this orbital is occupied and unoccupied respectively. Also, the difference in energy levels of the two spin states is almost 4 eV. In addition to ${\mathrm{d}}_{{\mathrm{z}}^{2}}$ (Fig. 3d) orbital, the ${\mathrm{d}}_{\mathrm{yz}}$ and ${\mathrm{d}}_{\mathrm{zx}}$ (Fig. 3c) orbitals of Mn are full in the up-spin state and vacant in the down-spin state. Hence, as shown in Fig. 2a, regarding the portion of 3d orbitals of Mn in the magnetic moment, it is observed that three orbitals of ${\mathrm{d}}_{{\mathrm{z}}^{2}}$, ${\mathrm{d}}_{\mathrm{yz}}$, and ${\mathrm{d}}_{\mathrm{zx}}$ involve in 30%, 26%, and 29% (in total 85%) of the obtained magnetic moment, respectively. Figure S1 of SI schematically presents the spin separation of these levels and deviation from the initial crystal field. According to PDOS diagram in Fig. 3e, the ${\mathrm{d}}_{\mathrm{xy}}$ and ${\mathrm{d}}_{{\mathrm{x}}^{2}-{\mathrm{y}}^{2}}$ orbitals have larger spatial extents. The filling pattern of up-spin and down-spin states of these two orbitals indicates that they have no considerable effect on the system magnetization. Therefore, as ${\mathrm{d}}_{{\mathrm{z}}^{2}},$ ${\mathrm{d}}_{\mathrm{yz}}$, and ${\mathrm{d}}_{\mathrm{zx}}$ are half-filled orbitals, the intensity of the magnetization for each up-spin state of these orbitals is almost $1 {\upmu }_{\mathrm{B}}$, which is $3 {\upmu }_{\mathrm{B}}$ overall. According to Fig. S2, the main contribution of 3d orbitals of Mn to the band structure, along with 3p orbitals of the S atom, reduces the band gap by 0.48 eV. For both Mn and S atoms, the up-spin orbitals in the valence band and down-spin orbitals in the conduction band are the main contributors to the VBM and CBM. These orbitals coincide with each other and are located at the $\Gamma$ point of the first Brillouin zone. Therefore, in the presence of Mn, the band gap reduces and changes to the direct gap. In the last subfigure of Fig. 3f, the spin density difference is presented for this case. Mn doping causes the electrons of the first-neighbor of S atom to have an opposite spin and be antiparallel relative to the Mn electrons. The level of polarization becomes negligible moving away from the impurity atom. Therefore, hybridization of d-p orbital, the electrons of 3p-orbital of the nearest S atom and of 3d-orbital of the Mn atom have opposite spins. To investigate the effect of atomic vacancy on the magnetic properties of the system in the presence of TM, the S atom which is located in position No. 1 in Fig. 1p is eliminated. As shown in Fig. 1p, eliminating the S atom causes the TM (position No. 3), Pd (Position No.2), and S atoms (first neighbor to No.1) to have a free or suspended band. If one S atom is diminished from the structure, the Mn and Pd atoms lose one less electron relative to the previous state. Therefore, in the presence of Mn impurity, the metal cation ${\mathrm{Mn}}^{+3}$ can have more than three electrons in the 3d level. According to Fig. 2a, the magnetization intensity of Mn without S vacancy is almost $2.995{\upmu }_{\mathrm{B}}$ and with S vacancy , it is $3.135{\upmu }_{\mathrm{B}}$, indicating a 0.14 ${\upmu }_{\mathrm{B}}$ increase relative to the former case. According to Fig. 3h, the spins of 3p-orbital of the S atom are symmetrical and have no significant magnetic property. The 3d orbitals have experienced some changes, of which the increased contribution of two planar orbitals ${\mathrm{d}}_{\mathrm{xy}}$ and ${\mathrm{d}}_{{\mathrm{x}}^{2}-{\mathrm{y}}^{2}}$ (Fig. 3k) to magnetization is one of the important changes. This result is obtained from comparing data of magnetic contribution in Fig. 2a and results presented in Fig. 2b for the percentage contribution of Mn orbitals to the magnetic moment. The contributions of these orbitals, respectively, are 8% and 7% in the absence of the S vacancy and 15% and 9% in the presence of vacancy. By removing the S atom, the system has lower planar symmetry relative to the pure state. Hence, the contribution of two planar orbitals ${\mathrm{d}}_{\mathrm{xy}}$ and ${\mathrm{d}}_{{\mathrm{x}}^{2}-{\mathrm{y}}^{2}}$ somewhat increases relative to the symmetric case. Based on data presented in Table S7, the band gap is entirely disappeared, and the structure becomes conductive by removing the S atom. Here, the role of 3d and 3p orbitals is also obvious so that VBM and CBM are almost tangential to the Fermi level at point Y, as shown in Figs. S2 and S3. It should be noted that, unlike the first case, down-spin orbitals in both valence and conduction bands play crucial roles. Albeit, around the valence band, the role of 3d orbital is of lower importance than that of 3p orbital.

Then we investigate the effect of removing a Pd atom, position No. 2 in Fig. 1r. By removing this atom, four first-neighbor S atoms of Pd will have free bonds. The magnetic energy ($\Delta {\mathrm{E}}_{\mathrm{spin}}$) arising from the Pd vacancy in the presence of Mn is equal to 501.11 meV as demonstrated in Table S5. In other words, the magnetic energy of ${\mathrm{Pd}}_{2}{\mathrm{S}}_{4}$ is also reduced relative to the initial and S vacancy states. The intensity of magnetization has also been mitigated by $0.472{\upmu }_{\mathrm{B}}$ relative to the case which there is no vacancy defect. The S atom, which is commonly bonded with Mn and Pd atoms, should gain more electrons from Mn by removing Pd. In this case, the magnetic moment is also reduced by $0.472{\upmu }_{\mathrm{B}}$, which means that the magnetic moment of adjacent S atoms increases, as presented in Table S6. It can also be found from spin density shown in Fig. 3r. The accurate value of this increase is presented in Tables S2 and S6 in the SI. According to Fig. 3m, which shows the band structure in the presence of Mn impurity and Pd vacancy, and Table S7 in the SI, there is no band gap in this case. As shown in the SI, Figs. S2 and S3, 3d orbitals of Mn atom and 3p orbitals of S atom cross the Fermi level. Regarding Fig. 3n, the 3p orbital of S atom and ${\mathrm{d}}_{\mathrm{yz}}$ and ${\mathrm{d}}_{\mathrm{zx}}$ (Fig. 3o) orbitals of Mn play crucial roles in cancelling the 0.73 eV band gap when only Mn atom is present as an impurity in the structure. The last figures of Fig. 3i,r, illustrate the difference of spin density in the presence of S and Pd vacancies, respectively, where the Mn doping in both of the cases causes the extension of polarity from the impurity atom to neighboring atoms. This polarization is more obvious when the Pd atom is removed from the system. Electrons of the 3p orbital of the nearest S atom have an opposite spin relative to electrons of the 3d orbital of the Mn atom.

### Electronic and magnetic properties of monolayer $\mathbf{P}{\mathbf{d}}_{2}{\mathbf{S}}_{4}$ in the presence of V atoms

**Figure 4:**
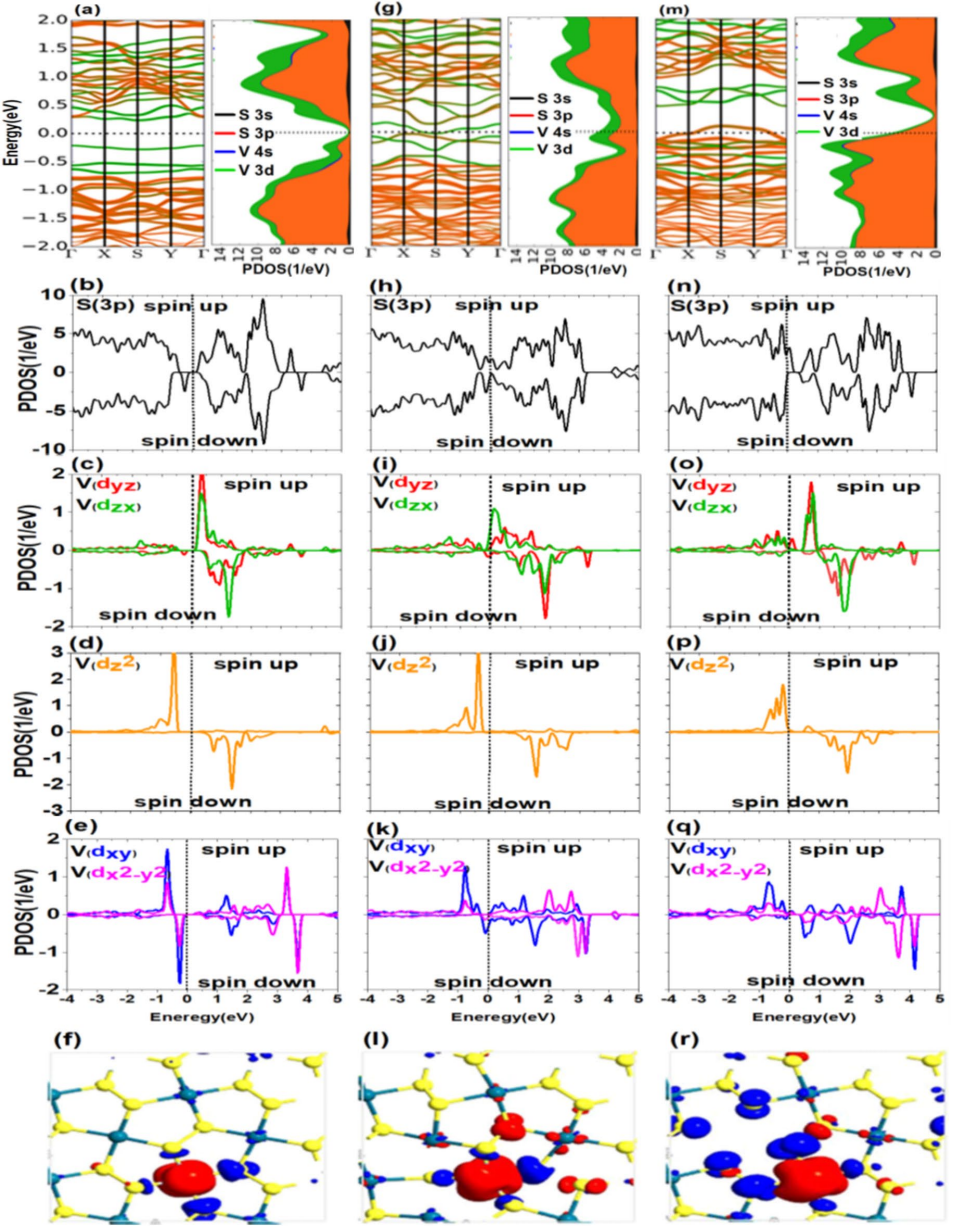
(**a–f**) Band structure and PDOS of p and d orbitals and spin density difference in the presence of transition metal impurity (V), (**g–i**) in the presence of the S atom defect, and (**m–r**) in the presence of the Pd atom defect.

In the following, the effect of adding of V element to ${\mathrm{Pd}}_{2}{\mathrm{S}}_{4}$ structure is examined, which have a moderate intensity of magnetization. When V atom with a valence configuration of $4{\mathrm{s}}^{2}3{\mathrm{d}}^{3}$ is substituted for a Pd atom in the structure, it must lose electrons as much as Pd atoms. According to Table S7 in the SI, in the presence of V atom the band gap of doped ${\mathrm{Pd}}_{2}{\mathrm{S}}_{4}$ is 0.44 eV which is decreased nearly by $0.76\mathrm{eV}$ with respect to the pure system. As shown in Fig. 4a–e, 3d orbitals of the V atom and 3p orbitals of the S atom have an excellent hybridization in the valence and conduction bands. The contributions of down-spin and up-spin orbitals are entirely specified in valence and conduction bands, respectively. Also, VBM entirely coincides with ${\varvec{\Gamma}}$ point, and CBM lies on point S in the first Brillouin zone. According to data presented in Fig. 2a, the intensity of magnetization of V is $1.395{\upmu }_{\mathrm{B}}$ when there are no S and Pd vacancy defects. As can be seen, this value is considerably lower than the first considered group. The 3p orbital of the S atom has no contribution to the magnetization of the structure thus the PDOS diagram for up-spin and down-spin states is almost symmetrical. Therefore, in this case, the major part of magnetization is due to the 3d orbital of V. Figure 2c clearly indicates that exchange interaction and, consequently, splitting of up and down spins of ${\mathrm{V}}^{+4}$ are lower than ${\mathrm{Mn}}^{+4}$. In this case, the maximum spin states separation is related to ${\mathrm{d}}_{{\mathrm{z}}^{2}}$ (Fig. 4d) as about $3\mathrm{eV}$. Moreover, one of the spin states is full, and the other is entirely vacant. Thus, a major part of $1.39{\upmu }_{\mathrm{B}}$ is related to this orbital. The residual value of magnetization intensity, equivalent to $0.39{\upmu }_{\mathrm{B}}$, is attributed to other orbitals. Therefore, due to the spin splitting, the primary perception that the magnetization of V atom is equal to the missed four electrons, cannot be true. The main point in doping with V and Co (according to Fig. 2c) is the considerable contribution of ${\mathrm{d}}_{{\mathrm{z}}^{2}}$ to the magnetization. In contrast to the relatively large contributions of three orbitals ${\mathrm{d}}_{{\mathrm{z}}^{2}}$, ${\mathrm{d}}_{\mathrm{yz}}$, and ${\mathrm{d}}_{\mathrm{zx}}$ (Fig. 2) to magnetization of the first group (Fe, Mn, and Cr), the major contribution for the second group is related to ${\mathrm{d}}_{{\mathrm{z}}^{2}}$ orbital. V and Co have 5 and 9 electrons in their valence band, respectively. Therefore, after being involved in the ${\mathrm{Pd}}_{2}{\mathrm{S}}_{4}$ structure, they lose four electrons and will have one (V) and five electrons (Co) in the 3d energy level. Hence, the single electron of V is placed in up-spin of  ${\mathrm{d}}_{{\mathrm{z}}^{2}}$ orbital, which is the reason why it has the most percentage of magnetization. Also, four electrons of Co with opposite spins occupy two orbitals of ${\mathrm{d}}_{\mathrm{yz}}$ and ${\mathrm{d}}_{\mathrm{zx}}$, and only the remaining one electron goes to ${\mathrm{d}}_{{\mathrm{z}}^{2}}$ orbital. There is no electron for Ti of valence shell $4{\mathrm{s}}^{2}3{\mathrm{d}}^{2}$ after being involved in the ${\mathrm{Pd}}_{2}{\mathrm{S}}_{4}$ structure.

According to Figs. S2 and S3 and Table S7 in the SI, the ${\mathrm{Pd}}_{2}{\mathrm{S}}_{4}$ structure becomes conductive because of S vacancy formation in the first neighbor of the doped structure. It can be found from Figs. S2 and S3 that Fermi level interrupts the 3d orbital of V and 3p orbital of S. Thus, 3d and 3p orbitals with up and down spins not only cross Fermi level but also interrupt each other at S point in the band structure due to the S vacancy defect. As illustrated in Fig. 4g–i and Tables S2 and S4 of the SI, the vacancy defect of S atom in the first neighborhood of V-doped ${\mathrm{Pd}}_{2}{\mathrm{S}}_{4}$ can increase the intensity of magnetization to $1.946{\upmu }_{\mathrm{B}}$ compared to the no vacancy one which is $1.39{\upmu }_{\mathrm{B}}$, where like the previous case, 3p orbitals (Fig. 4h) have fairly symmetrical shapes and no magnetization. In this case, the significant contribution of ${\mathrm{d}}_{{\mathrm{z}}^{2}}$ (Fig. 4j) to magnetization is clear. The high spin of ${\mathrm{d}}_{{\mathrm{z}}^{2}}$ (Fig. 4j) stays on the valence band, while this orbital has a vacant opposite spin. Also, the energy difference of up-spin and down-spin states under the effect of exchange interaction in ${\mathrm{d}}_{{\mathrm{z}}^{2}}$ is about 2.5 eV. For the remainder of magnetization, the other 3d orbitals must be considered. In the presence of vacancy defect at the first neighborhood (as shown in Fig. 2c), other orbitals have approximately the same contribution to the magnetization. According to Table S5 in the SI, the spin energy arising from the Pd vacancy defect in the V-dopped structure is − 432.72 meV. Also, based on Fig. 2a, the magnetic moment due to the Pd vacancy defect has increased about $0.70{\upmu }_{\mathrm{B}}$ compared to the pure system.

Regarding Figs. 2c and 4k, ${\mathrm{d}}_{{{\mathrm{x}}^{2}-\mathrm{y}}^{2}}$ and ${\mathrm{d}}_{\mathrm{xy}}$ have made a profound difference with three other orbitals ${\mathrm{d}}_{{\mathrm{z}}^{2}}$, ${\mathrm{d}}_{\mathrm{yz}}$, and ${\mathrm{d}}_{\mathrm{zx}}$ in terms of spin separation. Therefore, the main effect of increasing the magnetic moment compared to the pure system can be due to the effect of the new crystal field on these orbitals. Figures S2 and S3 and the magnitude of the band gap presented in Table S7 in SI show that the structure becomes conductive because of Pd atomic vacancy so that Fermi level shifts to the within of 3p (Fig. 4m,n) and 3d (Fig. 4m,o,p,q) orbitals. In the Fig. 4f,I,r, the spin density at impurity atom (V) has been illustrated for the structure without and with vacancies. The behavior of atoms adjacent to impurity in considered cases is similar to the the behavior of the atoms adjacent to the Mn impurity (see Fig. 3). The main difference is the lower intensity of magnetization in the presence of V, which the reasons were investigated above.

### Electronic and magnetic properties of monolayer $\mathbf{P}{\mathbf{d}}_{2}{\mathbf{S}}_{4}$ in the presence of Ni atoms

**Figure 5:**
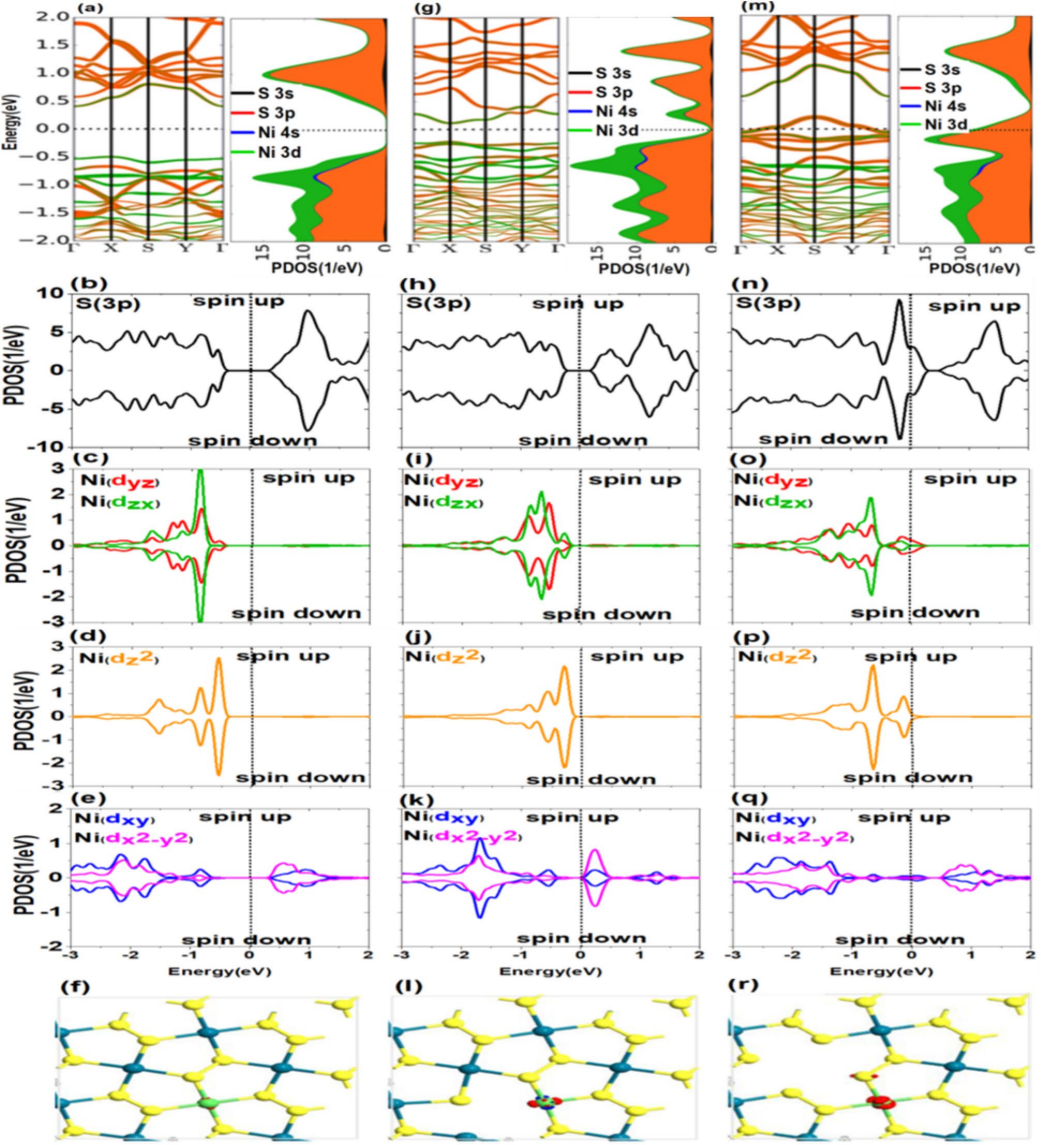
(**a–f**) Band structure and PDOS of p and d orbitals and spin density difference in the presence of transition metal impurity (Ni), (**g–i**) in the presence of the S vacancy, and (**m–r**) in the presence of the Pd vacancy.

When the Ni metal with valence configuration of $4{\mathrm{S}}^{2}3{\mathrm{d}}^{8}$ involves in the studied structure as the representation of the third group (Sc, Ni, Cu, and Zn), it shows no magnetic properties. The Ni atom has more three electrons than Mn, and in a straightforward comparison with the Mn atom, it is expected that these additional electrons can cover the entire magnetic moment obtained in the presence of Mn. As indicated in Fig. 2, Ni doping does not influence the magnetic properties of the intended system. The PDOS diagrams for up-spin and down-spin states are perfectly symmetrical and neutralize each other which can be referred to the nonmagnetic property. From Fig. 5a–f, it is observed that ${\mathrm{d}}_{{\mathrm{z}}^{2}}$(Fig. 5d) ${\mathrm{d}}_{\mathrm{yz}}$, and ${\mathrm{d}}_{\mathrm{zx}}$ (Fig. 5c) are within the valence band and fully occupied. Therefore, electrons of ${\mathrm{Ni}}^{+4}$ occupy orbitals of the lowest energy pairwise with opposite spins in a pair. Hence, there is no magnetization in this structure and the magnetic contribution of every component is zero. The role of Ni is similar to Pd because both of them have no spin separation and no magnetizing effect on the system. Similarly, Sc, Cu, and Zn also have symmetrical up- and down-spin densities and no spin separation. In this case, the required field for d orbitals splitting is the crystal field, which does not compete with exchange interaction for separating high-spin and low-spin states. Table S7 in the SI demonstrates that the change in band gap in the presence of Ni is less than the presence of Mn and V. According to Figs. S2 and S3 in SI, it can be stated that the structure still remains semiconductor when Ni is introduced, and the type of band gap remains indirect. VBM and CBM are coincident with ${\varvec{\Gamma}}$ point. The S vacancy in this structure leads to the band gap alteration but the system is non-magnetic (see Fig. 5h–l). In Table S7 of the SI, the band gap values arising from this defect are presented. From Figs. 5g, S2 and S3, it is found that the band gap is direct and VBM and CBM coincide with X point. The structure still remains nonmagnetic (see Fig. 5n–r) by removing the Pd atom and in the absence of exchange interaction, leading to the separation of spin states. In this case, the main difference is the intersection of Fermi level by p orbitals of S and ${\mathrm{d}}_{\mathrm{yz}}$ and ${\mathrm{d}}_{\mathrm{zx}}$ (Fig. 5m,o) orbitals of Pd. The addition of free electrons because of the S atom and initial symmetry elimination can be the reason for this behavior of energy level in altering the band gap in states with vacancy.

## Conclusion

This paper studied the development of magnetic properties and the electronic properties of monolayer ${\mathrm{Pd}}_{2}{\mathrm{S}}_{4}$ under the effect of doping with 3d transition metals with/without vacancy defects of S and Pd atoms. The crystal field decreases the degeneracy of d orbitals of 3d TM, and these orbitals are split approximately based on the square planar model. The addition of exchange interaction in the presence of 3d transition metals causes the new spin separations, especially in d orbitals. The spin splitting of ${\mathrm{d}}_{{\mathrm{z}}^{2}}$ is sometimes more considerable and effective in establishing the magnetic moment of the system where the spin energy difference of ${\mathrm{d}}_{{\mathrm{z}}^{2}}$ between up and down spins for Mn doping is almost 4 eV. This separation causes one of the spin states to be full and another one to be vacant. Calculations for all 3d TM of the periodic table indicate that doping with Mn, Cr, and Fe leads to the maximum intensity of magnetization in the structure. Some of 3d metals, such as Ti, V, and Co, cause less magnetization in the structure. Finally, doping with the 3d metals such as Sc, Ni, Cu, and Zn atoms, results in non-magnetic properties in the system. By applying the S and Pd atomic vacancies which are next to the TM impurities, the system symmetry mitigates, and the number of free electrons, which are previously in a bond with other atoms, increases. Therefore, the atomic vacancies, as obtained here, has no significant effect on the magnetic properties of the system and can directly affect the electronic properties such as the band gap.

## Supplementary Information

Supplementary Information.

## Data Availability

All data generated or analyzed during this study are included in this published article and its Supplementary Information flies which is available on the website of the journal.
