# Supplementary material for: Effects of 3d transition metal impurities and vacancy defects on electronic and magnetic properties of pentagonal Pd2S4: competition between exchange splitting and crystal fields
Source: Sci Rep. 2022 Jun 27;12:10838. doi: 10.1038/s41598-022-14780-z (PMC9237093; doi:10.1038/s41598-022-14780-z)
Supplement: Supplementary file 1 — Supplementary Information. [file 41598_2022_14780_MOESM1_ESM.docx]

**Effects of 3d transition metal impurities and vacancy defects on electronic and magnetic properties of pentagonal Pd_2_S_4_: Competition between exchange splitting and crystal fields**

Mojtaba gholami^,1.2^, Z. Golsanamlou^2^, H. Rahimpour Soleimani ^1,2 *^

^1^ Department of physics, University Campus2, University of Guilan, Rasht, Iran.

^2^ Computational Nanophysics Laboratory (CNL), Department of Physics, University of Guilan,

P. O. Box 41335‑1914, Rasht, Iran

* Corresponding authors: H. Rahimpour Soleimani (Rahimpour @guilan.ac.ir)

# **Supplementary information**

According to Tables S1 and S2, doping The initial elements of the 3d TM, such as$\mathrm{Sc}$, and the final elements, such as $\mathrm{Cu}$ and$\mathrm{Zn}$, do not play a role in creating magnetism in the ${{Pd}_{2}S}_{4}$ system. The reason for the lack of magnetization of the elements mentioned in the system has two different behaviors, which are examined following:

**Doping** $\mathbf{Sc}$

Sc atom with $3d^{1}4d^{2}$ atomic arrangement has 3 electrons in the valence shell, which loses four electrons when located in${{Pd}_{2}S}_{4}$ and appears as a cation $\mathrm{Sc}^{+4}$ in the system(Fig.1(p)). Thus $\mathrm{Sc}^{+4}$ not only lacks an electron in the $3d^{0}4d^{0}$ layer, but also loses one of the lower orbital electrons. Hence the doping-induced magnetization of$\mathrm{Sc}$ instead of palladium is absent due to the lack of electrons in the d orbital in the system. As shown in Fig.2(a), the$S$ and $\mathrm{Pd}$ defects cannot induce magnetism in the structure of${{Pd}_{2}S}_{4}$.

**Doping** $\mathbf{Cu , Zn}$

The metals Cu and Zn at the end of the 3d period are with 9 and 10 electrons in the valance layer, respectively. By doping the metals Cu and Zn in the structure of${{Pd}_{2}S}_{4}$, each of them loses 4 electrons as cations $\mathrm{Cu}^{+4}$ and $\mathrm{Zn}^{+4}$ and have 5 and 6 electrons, respectively. Therefore $\mathrm{Cu}^{+4}$ and $\mathrm{Zn}^{+4}$ have no magnetization despite having electrons in the d orbital. Unlike the Sc case, which lacks electrons in the valance layer, the lack of spin separation causes the structure to be nonmagnetic in the sentience of Cu and Zn metals. Also, based on the information in Tables S3, S5, and Fig. 2, it can be asserted that the S and Pd defects cannot induce magnetic in the structure.

**Table S1**. the magnetic and non-magnetic energy of 3d TM on the structure $\mathrm{Pd}_{2}S_{4}$

| **system** | $\mathbf{E}_{\mathbf{sp}}$**(eV)** | $\mathbf{E}_{\mathbf{nsp}}$**(eV)** | $\mathbf{E}_{\mathbf{spin}}$**(meV)** |
| --- | --- | --- | --- |
| Doping Sc | -31927.94299 | -31927.94300 | 0 |
| Doping Ti | -32224.60782 | -32224.45160 | -156.22 |
| Doping V | -32617.34739 | -32616.97719 | -370.2 |
| Doping Cr | -33088.04796 | -33087.30377 | -744.19 |
| Doping Mn | -33573.13992 | -33571.94515 | -1194.77 |
| Doping Fe | -34054.53417 | -34053.85887 | -675 |
| Doping Co | -34773.41298 | -34773.21611 | -196 |
| Doping Ni | -35174.96091 | -35174.96091 | 0 |
| Doping Cu | -35767.85676 | -35767.85676 | 0 |
| Doping Zn | -36297.29092 | -36297.29092 | 0 |

**Table S2** .The magnetic moment of the doping atom ($M_{d}$), four nearest neighboring $S$ atoms ($M_{s1}$) and $\mathrm{Pd}$ atoms ($M_{pd1}$) around dopant, other farther$S$ atoms ($M_{s2}$) and $\mathrm{Pd}$ atoms ($M_{pd2}$) for $\mathrm{Pd}_{2}S_{4}$ ($x=Sc ,Ti ,V,Cr,Mn,Fe ,Co, Ni ,Cu and Zn$).

| **system** | $\mathbf{M}_{\mathbf{d}}\mathbf{(}\boldsymbol{\mu}_{\mathbf{B}}\mathbf{)}$ | $\mathbf{M}_{\mathbf{s1}}\mathbf{(}\boldsymbol{\mu}_{\mathbf{B}}\mathbf{)}$ | $\mathbf{M}_{\mathbf{s2}}\mathbf{(}\boldsymbol{\mu}_{\mathbf{B}}\mathbf{)}$ | $\mathbf{M}_{\mathbf{pd1}}\mathbf{(}\boldsymbol{\mu}_{\mathbf{B}}\mathbf{)}$ | $\mathbf{M}_{\mathbf{pd2}}\mathbf{(}\boldsymbol{\mu}_{\mathbf{B}}\mathbf{)}$ |
| --- | --- | --- | --- | --- | --- |
| Doping Sc | 0.00 | 0.00 | 0.00 | 0.00 | 0.00 |
| Doping Ti | 0.70 | -0.084 | -0.002 | -0.01 | -0.002 |
| Doping V | 1.39 | -0.195 | -0.014 | -0.028 | -0.01 |
| Doping Cr | 2.433 | -0.087 | -0.039 | -0.002 | -0.014 |
| Doping Mn | 2.995 | -0.073 | 0.001 | 0.033 | -0.003 |
| Doping Fe | 2.036 | -0.098 | -0.041 | -0.001 | -0.016 |
| Doping Co | 0.93 | 0.009 | 0.015 | 0.005 | 0.005 |
| Doping Ni | 0.00 | 0.00 | 0.00 | 0.00 | 0.00 |
| Doping Cu | 0.00 | 0.00 | 0.00 | 0.00 | 0.00 |
| Dopping Zn | 0.00 | 0.00 | 0.00 | 0.00 | 0.00 |

With S defect. **Table S3.** the magnetic and non-magnetic energy of 3d TM on the structure $\mathrm{Pd}_{2}S_{4}$

| **defect type** | **1st neighbour S** | | |
| --- | --- | --- | --- |
| **system** | $\mathbf{E}_{\mathbf{sp}}$**(eV)** | $\mathbf{E}_{\mathbf{nsp}}$**(eV)** | $\mathbf{E}_{\mathbf{spin}}$**(meV)** |
| Sc | -31626.81502 | -31626.81502 | 0 |
| Ti | -31923.58499 | -31923.47532 | -109.67 |
| V | -32315.68351 | -32315.33200 | -351.51 |
| Cr | -32786.00516 | -32785.48955 | -515.61 |
| Mn | -33270.97521 | -33269.92150 | -1053.71 |
| Fe | -33752.31868 | -33751.68512 | -633.56 |
| Co | -34470.63566 | -34470.81488 | 179.22 |
| Ni | -34872.31779 | -34872.31779 | 0 |
| Cu | -35464.53321 | -35464.53321 | 0 |
| Zn | -35992.13616 | -35992.13616 | 0 |

**Table S4**.The magnetic moment of the doping and defect atom ($M_{d}$), four nearest neighboring $S$ atoms ($M_{s1}$*)* and $\mathrm{Pd}$ atoms ($M_{pd1}$) around dopant, other farther$S$ atoms ($M_{s2}$) and $Pd$ atoms ($M_{pd2}$) for $\mathrm{Pd}_{2}S_{4}$ ($x=Sc ,Ti ,V,Cr,Mn,Fe ,Co, Ni ,Cu and Zn$).

| **system** | $\mathbf{M}_{\mathbf{d}}\mathbf{(}\boldsymbol{\mu}_{\mathbf{B}}\mathbf{)}$ | $\mathbf{M}_{\mathbf{s1}}\mathbf{(}\boldsymbol{\mu}_{\mathbf{B}}\mathbf{)}$ | $\mathbf{M}_{\mathbf{s2}}\mathbf{(}\boldsymbol{\mu}_{\mathbf{B}}\mathbf{)}$ | $\mathbf{M}_{\mathbf{pd1}}\mathbf{(}\boldsymbol{\mu}_{\mathbf{B}}\mathbf{)}$ | $\mathbf{M}_{\mathbf{pd2}}\mathbf{(}\boldsymbol{\mu}_{\mathbf{B}}\mathbf{)}$ |
| --- | --- | --- | --- | --- | --- |
| Sc | 0.00 | 0.00 | 0.00 | 0.00 | 0.00 |
| Ti | 1.215 | -0.084 | -0.002 | -0.01 | -0.002 |
| V | 1.39 | -0.195 | -0.014 | -0.028 | -0.01 |
| Cr | 2.433 | -0.087 | -0.039 | -0.002 | -0.014 |
| Mn | 2.995 | -0.073 | 0.001 | 0.033 | -0.003 |
| Fe | 2.036 | -0.098 | -0.041 | -0.001 | -0.016 |
| Co | 0.93 | 0.009 | 0.015 | 0.005 | 0.005 |
| Ni | 0.00 | 0.00 | 0.00 | 0.00 | 0.00 |
| Cu | 0.00 | 0.00 | 0.00 | 0.00 | 0.00 |
| Zn | 0.00 | 0.00 | 0.00 | 0.00 | 0.00 |

**Table S5.** the magnetic and non-magnetic energy of 3d TM on the structure $\mathrm{Pd}_{2}S_{4}$ with Pd defect.

|  | |  | | | |
| --- | --- | --- | --- | --- | --- |
| **defect type** | | **1st neighbour Pd** | | | |
| **system** | | $\mathbf{E}_{\mathbf{sp}}$**(eV)** | $\mathbf{E}_{\mathbf{nsp}}$**(eV)** | $\mathbf{E}_{\mathbf{spin}}$**(meV)** | |
| Sc | | -28240.70008 | -28240.70008 | 0 | |
| Ti | | -28537.31597 | -28537.31601 | 0 | |
| V | | -28929.59082 | -28929.15810 | -432.72 | |
| Cr | | -29399.94644 | -29399.77558 | -170.86 | |
| Mn | | -29884.84341 | -29884.34230 | -501.11 | |
| Fe | | -30366.31462 | -30366.02823 | -286.39 | |
| Co | | -31085.12934 | -31084.99747 | -131.87 | |
| Ni | | -31486.46257 | -31486.46248 | 0 | |
| Cu | | -32079.50292 | -32079.50293 | 0 | |
| Zn | | -32608.82619 | -32608.82619 | 0 | |

**Table S6**.The magnetic moment of the doping and defect Pd atom ($M_{d}$), four nearest neighboring $S$ atoms ($M_{s1}$) and $\mathrm{Pd}$ atoms ($M_{pd1}$) around dopant, other farther$S$ atoms ($M_{s2}$) and $\mathrm{Pd}$ atoms ($M_{pd2}$) for $\mathrm{Pd}_{2}S_{4}$ ($x=Sc ,Ti ,V,Cr,Mn,Fe ,Co, Ni ,Cu and Zn$).

| **defect type** | **1st neighbour Pd** | | | | |
| --- | --- | --- | --- | --- | --- |
| **system** | $\mathbf{M}_{\mathbf{d}}\mathbf{(}\boldsymbol{\mu}_{\mathbf{B}}\mathbf{)}$ | $\mathbf{M}_{\mathbf{s1}}\mathbf{(}\boldsymbol{\mu}_{\mathbf{B}}\mathbf{)}$ | $\mathbf{M}_{\mathbf{s2}}\mathbf{(}\boldsymbol{\mu}_{\mathbf{B}}\mathbf{)}$ | $\mathbf{M}_{\mathbf{pd1}}\mathbf{(}\boldsymbol{\mu}_{\mathbf{B}}\mathbf{)}$ | $\mathbf{M}_{\mathbf{pd2}}\mathbf{(}\boldsymbol{\mu}_{\mathbf{B}}\mathbf{)}$ |
| Sc | 0 | 0 | 0 | 0 | 0 |
| Ti | 0 | 0 | 0 | 0 | 0 |
| V | 2.109 | -0.370 | -0.060 | -0.020 | -0.013 |
| Cr | 1.946 | -0.46 | -0.22 | -0.070 | -0.040 |
| Mn | 2.523 | -0.360 | -0.30 | -0.060 | -0.040 |
| Fe | 2.12 | 0.23 | 0.010 | 0.040 | -0.015 |
| Co | 1 | 0.33 | 0.030 | 0.035 | 0.013 |
| Ni | 0 | 0 | 0 | 0 | 0 |
| Cu | 0 | 0 | 0 | 0 | 0 |
| Zn | 0 | 0 | 0 | 0 | 0 |


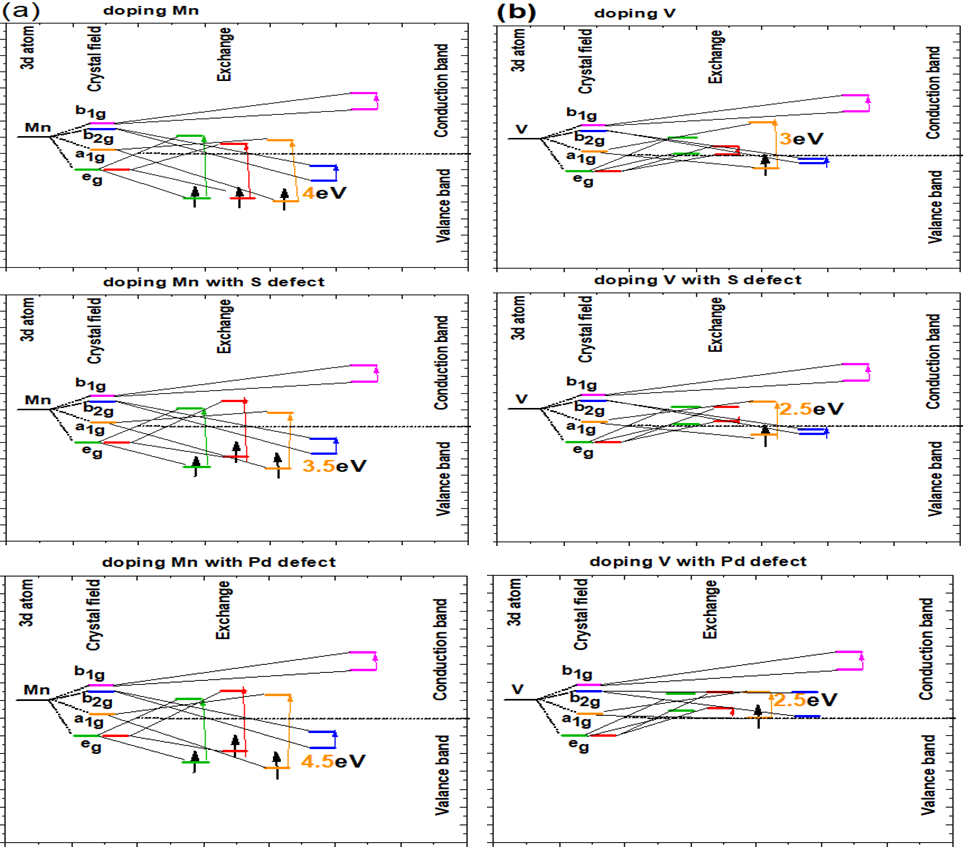


**Figure S1**. Schematic of spin separation of 3d TM (a) Mn orbitals without defect and with defect of S and Pd atoms, (b) V without defect and with defect of S and Pd atoms.

**Table S7**. The gap energy in the presence of TM doping and S and Pd defects in the monolayer Pd2S4 system.

| **defect type** | **Without atomic vacancy** | **1st neighbour S** | **1st neighbour Pd** |
| --- | --- | --- | --- |
| **Doping** | $\mathbf{E}_{\mathbf{g}}$**(eV)** | $\mathbf{E}_{\mathbf{g}}$**(eV)** | $\mathbf{E}_{\mathbf{g}}$**(eV)** |
| Sc | 1 | 0.44 | 0.67 |
| Ti | 0.18 | 0 | 0.35 |
| V | 0.44 | 0 | 0 |
| Cr | 0.35 | 0.33 | 0 |
| Mn | 0.73 | 0.14 | 0.3 |
| Fe | 0.27 | 0.22 | 0 |
| Co | 0.44 | 0.27 | 0 |
| Ni | 0.90 | 0.33 | 0.37 |
| Cu | 0.45 | 0.44 | 0.74 |
| Zn | 1.1 | 0.52 | 0.85 |


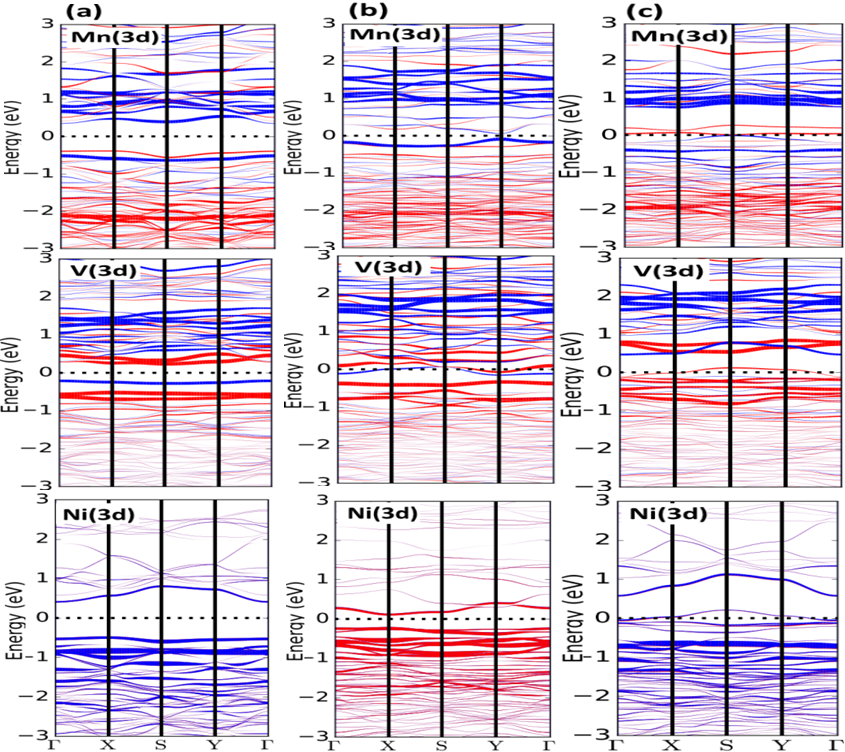


**Figure S2**. bandstructure of Pd-3d orbital in the presence of TM doping (a) Mn, V and Ni without defect (b) in the S defect with doping Mn, V and Ni (c) in the Pd defect with doping Mn, V and. (The up-spin and down spin are shown in red and blue, respectively).


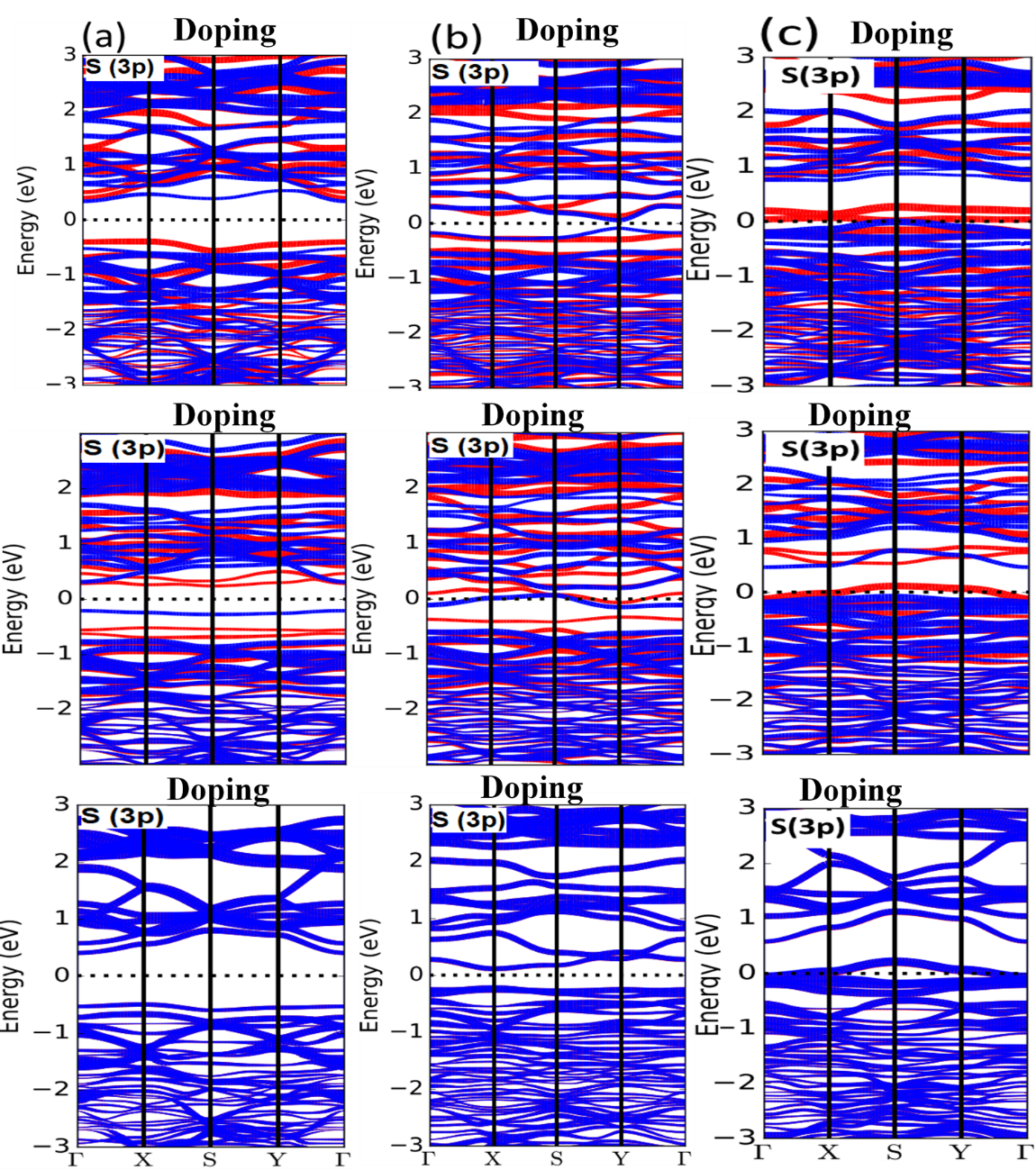


**Figure S3**. bandstructure of S-3p orbital in the presence of TM doping (a) Mn, V and Ni without defect (b) in the S defect with doping Mn, V and Ni (c) in the Pd defect with doping Mn, V and. (The up-spin and down spin are shown in red and blue, respectively).
